# Whey Protein Supplementation Post Resistance Exercise in Elderly Men Induces Changes in Muscle miRNA's Compared to Resistance Exercise Alone

**DOI:** 10.3389/fnut.2019.00091

**Published:** 2019-06-12

**Authors:** Randall F. D'Souza, Nina Zeng, James F. Markworth, Vandre C. Figueiredo, Christopher P. Hedges, Aaron C. Petersen, Paul A. Della Gatta, David Cameron-Smith, Cameron J. Mitchell

**Affiliations:** ^1^Liggins Institute, The University of Auckland, Auckland, New Zealand; ^2^Discipline of Nutrition, The University of Auckland, Auckland, New Zealand; ^3^Department of Molecular and Integrative Physiology, University of Michigan, Ann Arbor, MI, United States; ^4^College of Health Sciences, University of Kentucky, Lexington, KY, United States; ^5^Applied Surgery and Metabolism Laboratory, School of Biological Sciences, University of Auckland, Auckland, New Zealand; ^6^Institute for Health and Sport (iHeS), Victoria University, Melbourne, VIC, Australia; ^7^Institute for Physical Activity and Nutrition (IPAN), School of Exercise and Nutrition Sciences, Deakin University, Geelong, VIC, Australia; ^8^Food and Bio-based Products, AgResearch Grasslands, Palmerston North, New Zealand; ^9^The Riddet Institute, Massey University, Palmerston North, New Zealand; ^10^School of Kinesiology, University of British Colombia, Vancouver, BC, Canada

**Keywords:** skeletal muscle, mTOR pathway, microRNA, older adults, resistance training, P70S6 K, protein dose

## Abstract

Progressive muscle loss with aging results in decreased physical function, frailty, and impaired metabolic health. Deficits in anabolic signaling contribute to an impaired ability for aged skeletal muscle to adapt in response to exercise and protein feeding. One potential contributing mechanism could be exerted by dysregulation of microRNAs (miRNAs). Therefore, the aim of this study was to determine if graded protein doses consumed after resistance exercise altered muscle miRNA expression in elderly men. Twenty-three senior men (67.9 ± 0.9 years) performed a bout of resistance exercise and were randomized to consume either a placebo, 20 or 40 g of whey protein (*n* = 8, *n* = 7, and *n* = 8, respectively). *Vastus lateralis* biopsies were collected before, 2 and 4 h after exercise. Expression of 19 miRNAs, previously identified to influence muscle phenotype, were measured via RT-PCR. Of these, miR-16-5p was altered with exercise in all groups (*p* = 0.032). Expression of miR-15a and-499a increased only in the placebo group 4 h after exercise and miR-451a expression increased following exercise only in the 40 g whey supplementation group. Changes in p-P70S6K^Thr389^ and p-Akt^Ser473^ following exercise were correlated with alterations in miR-208a and-499a and-206 expression, irrespective of protein dose, suggesting a possible role for miRNA in the regulation of acute phosphorylation events during early hours of exercise recovery.

## Introduction

Progressive muscle loss with aging results in decreases in physical function, frailty and metabolic health (1–3). Age-related impairment in the responses to anabolic stimuli such as protein ingestion and resistance exercise contribute to declining muscle mass (4, 5). Deficits in the activation of anabolic signaling proteins play a role in the anabolic resistance to protein feeding (6), however, the regulatory mechanisms are still unclear. MicroRNAs (miRNAs), small non-coding RNAs, regulate gene expression via promotion of transcript breakdown and inhibition of protein translation (7, 8). Recent evidence suggests that miRNA may play a role in the mechanism of anabolic resistance and regulation of cellular phosphorylation events (9, 10).

It is widely accepted that miRNAs regulate cellular control via transcriptional and translation inhibition. There is also emerging evidence that specific miRNAs including miR-499a,-208a, -206,-133a,-1,-99a, 99b,-100, and-149 are involved in controlling intramuscular signaling of key proteins including Akt, P70S6K, and RPS6. These proteins are critical mediators of anabolic signaling via the Akt-mTOR pathway and thus upstream regulators of muscle protein synthesis (MPS) (8–11). The ability of miRNAs to impact kinase phosphorylation *in vitro* has also been demonstrated (12). In cell models, miRNAs alter anabolic signaling, but it is unclear if this occurs as a result of direct regulation of kinase phosphorylation or indirectly via control of upstream gene expression influencing posttranslational modifications (12). As yet, no relationship *in-vivo* between the phosphorylation status of proteins such as Akt and P70S6K and the expression of these proposed miRNA regulators has been identified.

Zacharewicz et al. (9), identified via PCR validation of a microarray analysis, seven miRNAs that were differentially altered following exercise in young compared to old men while Rivas et al. identified 21 miRNAs regulated by exercise in young men but not old men (12). Only a single study (13) has assessed both the resting and post resistance exercise muscular miRNA abundances following protein supplementation in young and elderly males. In this study, Drummond et al. reported increased expression of miR-1 and the immature form of miR-133a (pri-miRNA) in elderly compared to young men at rest (13). Following exercise in combination with ingestion of 20 g essential amino acid (EAA), miR-1 was reduced in muscle of young but not elderly men (13). In young adults, protein but not placebo ingestion after concurrent exercise reportedly altered miRNA expression from rest at 4 h following exercise (14). Little is known about how protein ingestion might alter the acute response of miRNAs more recently identified as regulators of muscle growth to resistance exercise, especially in aged muscle.

The primary aim of the current study was to characterize muscular responses of putative miRNA regulators of muscle anabolism to acute resistance exercise in older men and whether graded amounts of whey protein ingestion alters that response. The secondary aim of the study was to identify whether changes in miRNA expression following exercise correlated with changes in Akt-mTOR pathway phosphorylation status in agreement with the relationships proposed by Margolis and Rivas (10). It is hypothesized that whey protein ingestion will alter miRNA expression patterns following exercise in a dose dependent fashion. Further, as suggested by Margolis and Rivas (10), a relationship between changes in Akt-mTOR phosphorylation status and miRNA expression will observed.

## Methods

### Participants

Twenty-three elderly men (>60 years of age) from a larger clinical trial of sixty participants were included in the current study (Table 1) (15). Individuals who were undertaking any regular resistance exercise training or those with pre-existing metabolic or cardiovascular diseases were excluded. Participants who were taking anticoagulation or antihypertensive medications were not excluded from participation. All participants were recreationally active and completed at least 150 min per week of moderate intensity physical activity (walking, cycling, golf) and no more than 90 min a week of vigorous activity (jogging, faster cycling). Subjects taking aspirin/fish oil supplements were required to abstain from these medications throughout the duration of the trial. Prior to commencement of the study, participants were provided with written and oral information regarding the experimental protocols and potential risks involved and written consent to participate was obtained. All experimental procedures employed by this study were carried out in coherence with the Helsinki declaration and were formally approved by the Deakin University Human Research Ethics Committee.

**Table 1:** Participant characteristics.

	**Placebo (*n =* 8)**	**20 g whey (*n =* 7)**	**40 g whey (*n =* 8)**
Age (years)	67.3 ± 4.0	69.3 ± 3.7	67.4 ± 4.0
Height (cm)	180 ± 8	178 ± 11	181 ± 6
Weight (kg)	87.5 ± 14.7	90.5 ± 19.6	86.4 ± 12.2
BMI (kg/m^2^)	27.2 ± 5.1	28.6 ± 5.3	26.5 ± 3.7
Thigh CSA (cm^2^)	109.7 ± 16.7	120.6 ± 65.3	105.7 ± 44.6
1-RM Squat (kg)	73.9 ± 31.7	81.0 ± 27.0	91.9 ± 48.6
1-RM Leg Press (kg)	196.9 ± 80.0	211.9 ± 112.7	215.5 ± 71.3
1-RM Leg Ext.(kg)	49.5 ± 24.3	49.9 ± 29.9	64.2 ± 35.6

*Values presented as means ± SD. 1-RM values were estimated via the Brzycki equation*.

### Experimental Protocol

At least 1 week prior to the experimental trial day, a familiarization session which included one-repetition maximum (1RM) strength testing to determine the experimental exercise load (80% of 1RM) was conducted. The maximal weight that subjects could lift for 3–6 repetitions (3–6RM) of bilateral smith machine barbell squat, 45° leg press and seated leg extension exercises was determined and participants' 1RM was estimated using the Brzycki equation (16). In the week prior to the trial day, participants were instructed to abstain from any vigorous physical activity (>6 Mets). The evening before the trial, participants ingested a standard evening meal (2103 kJ, 54% carbohydrate, 29% fat, 17% protein) before 10 pm and were instructed to eat nothing afterward. The following morning, the subjects arrived (~7 a.m.) at the lab in a fasted state. Participants were randomly allocated into one of three treatment groups; non-caloric placebo (*n* = 8), 20 g whey (*n* = 7), and 40 g whey (*n* = 8).

### Determination of Thigh CSA

Thigh CSA was determined via anthropometric measurement as described previously (17). The formula used for calculation was

$$\begin{array}{r} A_{M}\;=\;0.649\;\times \left({\left[\frac{C_{T}}{\pi \;-\;S_{Q}}\right]}^{2}\;-{\left(0.3\;\times \;d_{E}\right)}^{2}\right) \end{array}$$

where: A_M_ = Mid-thigh muscle cross sectional area (cm^2^)

C_T_ = Thigh circumference (cm)

S_Q_ = Skinfold thickness of anterior quadriceps (cm)

d_E_ = distance across the medial and lateral femoral epicondyle (cm).

### Resistance Exercise and Supplementation Trial

Upon arrival at the laboratory, individuals rested in a supine position for ~30 min prior to collection of resting muscle biopsy samples (see below). Participants then rested supine following collection of resting muscle biopsy for approximately ~10–15 min after which the exercise protocol commenced. The exercise protocol began with a 10-min warm-up involving light cycling on a bicycle ergometer and a single low load warm-up set for each of the three exercises. Participants then completed three sets of 8–10 repetitions of bilateral barbell smith rack squat, 45° leg press, and seated knee extensions at 80% of their predetermined 1RM. Exercises were performed in a circuit manner with 1 min rest between each exercise and 3 min rest between subsequent sets, the exercise protocol took ~20 min to complete. Following the exercise protocol, subjects were immediately provided with a beverage, containing a non-caloric placebo, or one of the two doses of whey protein concentrate (WPC instantized 8010, Hilmar Ingredients, Hilmar, CA, USA [20 g, or 40 g]) dissolved in 350 mL of water. All supplements were vanilla flavored and sweetened with aspartame. Amino acid composition of the protein supplement is presented in the Table 2. Subjects were instructed to ingest the beverage within 2 min following which they were rested in a supine position throughout the 4 h of post-exercise recovery with additional muscle biopsy samples collected at 2 and 4 h post exercise. A whey protein dose of 20 g was chosen because it has been shown to maximize post exercise anabolism in young men (18) while a 40 g group was included because this dose provokes a larger post exercise MPS and signaling response in older men (4, 15).

**Table 2:** Amino acid profile of the whey protein supplement utilized in the current study.

**Amino acids represented as g/100 g of product**	
Alanine	4.2
Arginine	2.2
Aspartic Acid	8.7
Cystine	2.0
Glutamic Acid	14.0
Glycine	1.5
Histidine	1.5
Hydroxyproline	<0.1
Isoleucine^*^^$^	5.2
Leucine^*^^$^	8.5
Lysine^*^	7.8
Methionine^*^	1.8
Phenylalanine^*^	2.6
Proline	4.9
Serine	4.1
Threonine^*^	5.7
Tryptophan^*^	1.7
Tyrosine	2.5
Valine^*^^$^	4.5

* *Essential Amino Acids*.

$ *Branched-Chain Amino Acids (BCAA)*.

### Muscle Biopsy Sampling

Muscle biopsies (~100 mg) were collected from the *vastus lateralis* muscle under local anesthesia (1% Xylocaine) using a Bergstrom needle modification of manual suction. All three biopsies were collected from the same limb starting distally and moving proximally. A gap of at least 2–3 cm between sequential biopsies was maintained to avoid potential confounding effects caused by repeated sampling from the same location. Biopsies were quickly frozen in liquid nitrogen and stored at -80°C until further analyses.

### Muscle miRNA Isolation and RT/PCR

As previously described (8, 19) total RNA was extracted from ~20 mg of muscle tissue using the AllPrep® DNA/RNA/miRNA Universal Kit (QIAGEN GmbH, Hilden, Germany). Ten nanogram of total RNA from muscle was used for cDNA synthesis using TaqMan™ Advanced miRNA cDNA Synthesis Kit (Thermo Fisher Scientific, Carlsbad, CA, USA) and miRNA abundance were measured by RT-PCR on a QuantStudio 6 (Thermo Fisher Scientific, Carlsbad, CA, USA) using Applied Biosystems Fast Advanced Master Mix (Thermo Fisher Scientific, Carlsbad, CA, USA).

Target miRs are shown in Table 3 (Thermo Fisher Scientific, Cat# A25576, Carlsbad, CA, USA). The geometric mean of three reference miRs (miR-361,-320a, and-186) for muscle (20) were used for normalization based on miRs that showed the least variation amongst the current sample set. The mean CTs ± CV% for each reference miRNA was 23.73 ± 4.23%, 23.63 ± 4.45%, and 24.40 ± 3.91%. RT-PCR data was analyzed using 2^−ΔΔ*CT*^ method (21). Fold changes are reflective of each participants response compared to their individual pre-exercise values.

**Table 3:** Catalog numbers for the miRNAs analyzed and housekeepers with Thermo Fisher Scientific independent miR assay IDs.

**miR**	**ID Number**
miR-15a-5p	477858_mir
miR-16-5p	477860_mir
miR-23a-3p	478532_mir
miR-23b-3p	478602_mir
miR-451a	477968_mir
miR-486-5p	478128_mir
miR-126-3p	477887_mir
miR-133a-3p	478511_mir
miR-206	477968_mir
miR-1-3p	477820_mir
miR-148b-3p	477806_mir
miR-30b-5p	478007_mir
miR-145-5p	477916_mir
miR-499a-3p	478948_mir
miR-100-5p	478224_mi
miR-99a-5p	478519_mir
miR-149-5p	477917_miR
miR-208a-3p	477819_mir
miR-186-5p	477940_mir
miR-320a	478594_mir
miR-361-5p	478056_mir

### Western Blotting

Approximately 50 mg of muscle tissue was homogenized in ice-cold RIPA containing protease and phosphatase inhibitors (15). Homogenates were agitated for 1 h at 4°C and centrifuged for 15 min at 13,000 g. Protein content was determined using a BCA-protein assay kit (Pierce, Rockford, IL) according to the manufacturer's instructions. Aliquots of protein homogenate containing 50 μg of total protein were prepared, mixed with Laemmli buffer, boiled, and subjected to SDS/PAGE. Proteins were separated on an 8% gel and wet-transferred to a polyvinyl difluoride (PVDF) membrane. Following transfer, membranes were blocked in 5% bovine serum albumin (BSA)/Tris Buffered Saline/0.1% Tween 20 (TBST) for 1 h, followed by overnight incubation at 4°C with primary antibody against p-p70S6K (Thr389; 1:1,000, Cell Signaling, Danvers, MA) and p-Akt (Ser473, 1:1,000, cell signaling, Danvers, MA). p-AKT^Ser473^ was normalized to total Akt (Total Akt Cell Signaling 1:1,000). Due to large changes observed in the electrophoretic mobility of the Total p70S6K protein in highly phosphorylated postexercise and supplementation muscle samples it was difficult to accurately quantify total p70S6K. This large magnitude mobility shift in total p70S6K in samples with large degrees of p70S6K phosphorylation has been previously described (22). Total ERK2 (ERK1/2 Cell Signaling, 1:1,000) was thus used as a loading control because it did not change in any condition (15). Once normalized to a total, protein expression was also normalized to a pooled control sample loaded onto every gel so a correlation analysis could be performed.

### Statistical Analysis

Two way repeated measures analysis of variance with time as a within subject factor and group as a between subject factor was conducted using SigmaPlot for Windows version 12.1 (Systat 218 Software Inc., San Jose, USA) to determine differences in miRNA expression. Where appropriate, group and time differences were assessed using Holm–Sidak *post hoc* tests. Based on putative relationships suggested by Margolis and Rivas (10), independent Pearson correlations were assessed between p-P70S6K^Thr389^ and miR-208a and miR-499a. Similarly, p-Akt^Ser473^ expression was correlated with miR-208a, 206,-133a,-499a, and-100. A Pearson correlation was assessed between resting miR-133a expression and thigh muscle CSA based on our previous findings where muscle mass and size measures were negatively related to miR-133a (23–25). Correlations were assessed using SigmaPlot for Windows version 12.1 (Systat 218 Software Inc., San Jose, USA). Data are shown as mean ± SD unless specified. Statistical significance was accepted at *p* < 0.05. Figures were drawn using GraphPad Prism 7 Software (GraphPad Software Inc., La Jolla, CA).

## Results

**Figure 1:**
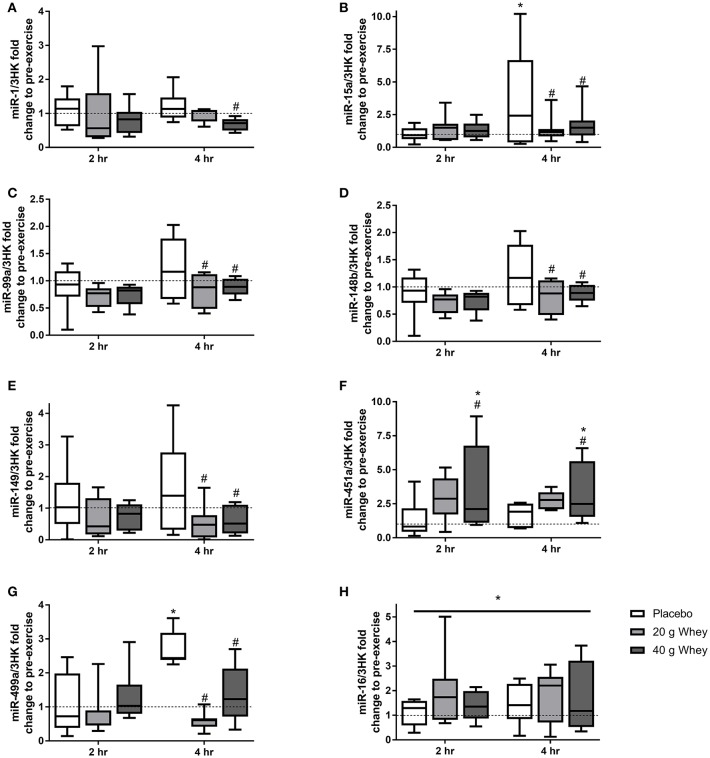
miRNA expression. Fold changes of (**A**) miR-1, (**B**) miR-15a, (**C**) miR-99a, (**D**) miR-148b, (**E**) miR-149, (**F**) miR-451a, (**G**) miR-499a, and (**H**) miR-16 in placebo, 20 g and 40 g whey groups at 2 hr and 4 hr following exercise. ^*^Difference between respective pre exercise expression *P* < 0.05. #Difference compared to placebo group at respective post-exercise time point, *P* < 0.05. Data are expressed as median ±1–99% confidence intervals as a fold change to respective pre-exercise expression. The boxes depict interquartile ranges. Dotted line reflects pre-exercise expression levels.

Expression of 19 miRNAs were assessed before, 2 and 4 h following the resistance exercise and feeding stimuli. Seven miRNAs (miR-1,-15a,-99a,-148b,-149,-451a, and-499a) demonstrated group by time interactions (*p* = 0.048, *p* = 0.006, *p* = 0.025, *p* = 0.043, *p* = 0.039, *p* = 0.036, and *p* = 0.047, respectively) (Figure 1). miR-15a was elevated at 4 h compared to rest (*p* = 0.013), a change that was attenuated in the 20 and 40 g protein groups (*p* < 0.001 and *p* = 0.003, respectively). Similarly, miR-499a was increased at 4 h after exercise in the placebo group (*p* = 0.027) and this response was suppressed in both the 20 and 40 g whey groups (*p* = 0.001 and *p* = 0.002, respectively). Also, at 4 h post-exercise miR-99a,-148b, and-149 exhibited lower expression in the 20 g protein group (*p* = 0.019, *p* = 0.011, and *p* = 0.017, respectively) and the 40 g protein group (*p* = 0.041, *p* = 0.011, and *p* = 0.19, respectively) when compared to the placebo group (Figures 1C–E). However, miR-99a,-148b, and -149 abundance were not altered following exercise in the placebo group (*p* = 0.247, *p* = 0.166, and *p* = 0.222). miR-1 was downregulated in the 40 g but not 20 g whey group when compared to the placebo group at 4 h following exercise (*p* = 0.023 and *p* = 0.193, respectively) (Figure 1A).

Muscle miR-451a appeared increase following exercise with only the 40 g whey group at both 2 and 4 h following exercise when compared to rest (*p* = 0.0063 and *p* = 0.0191, respectively) (Figure 1F). miR-16 demonstrated no group by time interaction however, there was a main effect of time *p* = 0.0327 (Figure 1H). Data and *p*-values for 10 additional miRNAs measured which did not exhibit significant group by time interaction effects are presented in Table 4.

**Table 4:** Fold change of muscle miRNA expression in each group following exercise compared to respective resting values.

	**Placebo**		**20 g Whey**		**40 g Whey**		
**miR**	**2 h**	**4 h**	**2 h**	**4 h**	**2 h**	**4 h**	***p*-value**
miR-23a	0.92 ± 0.65	0.88 ± 0.48	1.49 ± 1.29	1.15 ± 0.58	1.23 ± 1.22	1.25 ± 1.16	0.858
miR-23b	0.95 ± 0.88	0.78 ± 0.40	1.11 ± 0.56	1.05 ± 0.85	1.25 ± 0.79	1.27 ± 0.71	0.915
miR-30b	1.20 ± 0.57	1.14 ± 0.54	1.16 ± 0.66	1.32 ± 1.03	1.05 ± 0.79	1.07 ± 0.51	0.968
miR-100	1.13 ± 0.71	1.15 ± 0.85	0.76 ± 0.11	1.20 ± 1.48	0.65 ± 0.23	1.33 ± 0.85	0.725
miR-126	1.29 ± 0.76	1.10 ± 0.88	1.40 ± 0.61	1.38 ± 0.98	1.17 ± 0.65	1.54 ± 0.71	0.762
miR-133a	1.55 ± 1.24	1.60 ± 1.84	1.90 ± 1.03	2.16 ± 2.14	1.65 ± 2.52	2.15 ± 1.64	0.979
miR-145	1.10 ± 0.65	0.95 ± 0.65	0.98 ± 0.48	1.10 ± 0.93	1.12 ± 0.71	1.32 ± 0.88	0.899
miR-206	1.39 ± 1.30	0.99 ± 0.68	1.48 ± 0.82	1.42 ± 0.85	1.14 ± 0.45	0.93 ± 0.57	0.829
miR-208a	1.46 ± 1.27	2.07 ± 2.12	1.23 ± 1.24	1.56 ± 2.01	1.17 ± 0.65	1.50 ± 0.82	0.969
miR-486	1.01 ± 0.34	1.35 ± 1.19	1.30 ± 0.85	1.32 ± 0.95	1.13 ± 0.48	1.26 ± 0.83	0.966

**Figure 2:**
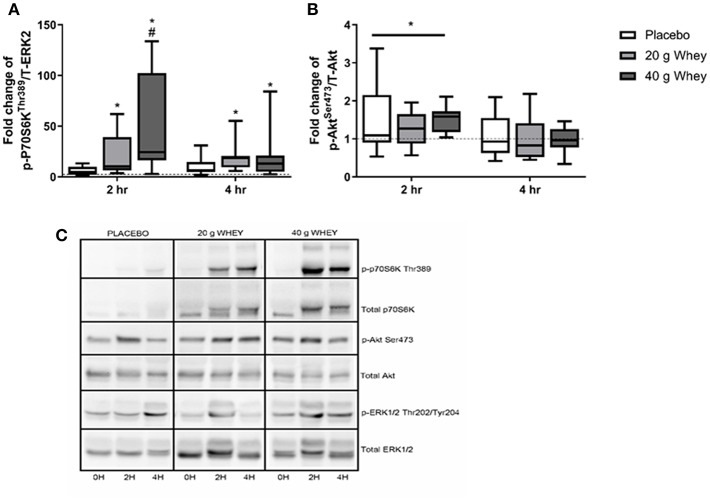
Phosphoprotein expression. Fold changes of **(A)** p-P70S6k^Thr389^ and **(B)** p-Akt^Ser473^in placebo, 20 g and 40 g whey groups at 2 h and 4 h following exercise. **(C)** Shows representative western blot images. *Difference between respective pre-exercise expression, *P* < 0.05. #Difference compared to placebo group at respective post-exercise time point, *P* < 0.05. Data are expressed as median ±1–99% confidence intervals as a fold change to respective pre-exercise expression. The boxes depict interquartile ranges. Dotted line reflects pre-exercise expression levels. Data for p-P70S6k^Thr389^ was originally published for a larger cohort (15).

*Values presented as means ± SD, n = 8 placebo, n = 7, 20 g whey, and n = 8, 40 g whey. p-values represent group by time interactions from two-way repeated measures ANOVA*.

p-P70S6K^Thr389^ has previously been reported for a larger cohort of participants including those in the present study (15). Immunoblotting demonstrated a group by time interaction for p-P70S6K^Thr389^ (*p* < 0.001) with only the 40 g whey group being elevated compared to the placebo group at 2 h post exercise (*p* < 0.001) (Figures 2A,C). At 2 h abundance of p-P70S6K^Thr389^ in the 20 and 40 g group was elevated and compared to pre-exercise (*p* = 0.036 and *p* < 0.001, respectively). This elevation was also evident in both groups at 4 h rfollowing exercise (*p* = 0.046 and *p* = 0.008, respectively). p-Akt^Ser473^ abundance demonstrated a main effect of time (*p* = 0.001) but no group by time interaction (*p* = 0.745). p-Akt^Ser473^ abundance was significantly elevated following exercise at 2 h and returned to pre-exercise levels by 4 h following exercise (*p* < 0.001 and *p* = 0.574) (Figures 2B,C). p-Akt^Ser473^ abundance at 2 h in all groups was also increased compared to abundance at 4 h (*p* < 0.001).

**Figure 3:**
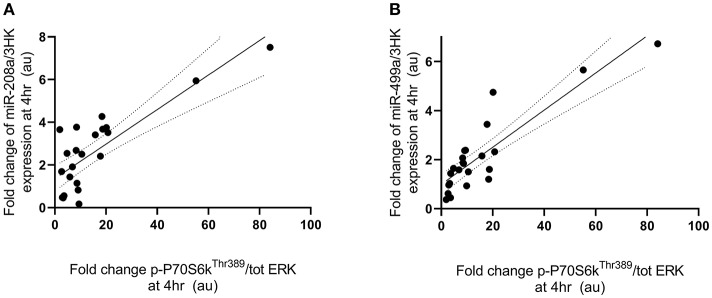
miRNAs correlated with post-exercise P70S6K^Thr389^ phosphorylation. (**A**) miR-208a and (**B**) miR-499a. miRNAs are plotted as a fold change from pre-exercise on the y-axis with fold change of p-P70S6K^Thr389^/ERK1/2 on the x-axis. The solid line represents the line of best fit as determined by linear regression with 95% confidence intervals.

**Figure 4:**
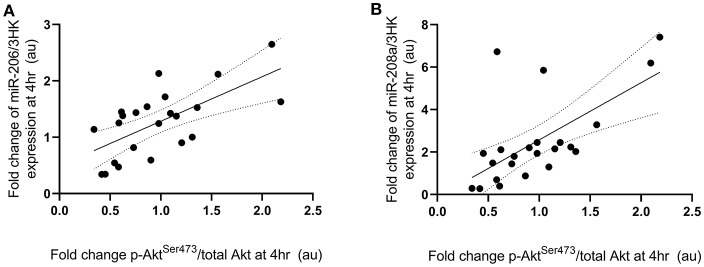
miRNAs significantly correlated with post-exercise Akt^Ser473^ phosphorylation. (**A**) miR-206 and (**B**) miR-208a. miRNAs are plotted as a fold change from pre-exercise on the y-axis with fold change of p-Akt^Ser473^/total Akt on the x-axis. The solid line represents the line of best fit as determined by linear regression with 95% confidence intervals.

Fold change in p-P70S6K^Thr389^ expression at 4 h following exercise was positively correlated with the fold change in miR-208a and -499a expression at 4 h post-exercise (*p* < 0.001 and *R*^2^ = 0.680 and *p* < 0.001 and *R*^2^ = 0.722, respectively) (Figures 3A,B). p-Akt^Ser473^ expression at 4 h following exercise was positively correlated with miR-206 (*p* = 0.001 and *R*^2^ = 0.461) and miR-208a (*p* < 0.001 and *R*^2^ = 0.628) (Figures 4A,B). Inconsistent with Margolis and Rivas (10), no relationships between changes in p-Akt^Ser473^ and miR-133a,-499a, and -100 expression were identified (*p* = 0.735 and *R*^2^ = 0.008, *p* = 0.808 and *R*^2^ < 0.001, and *p* = 0.634 and *R*^2^ = 0.020).

**Figure 5:**
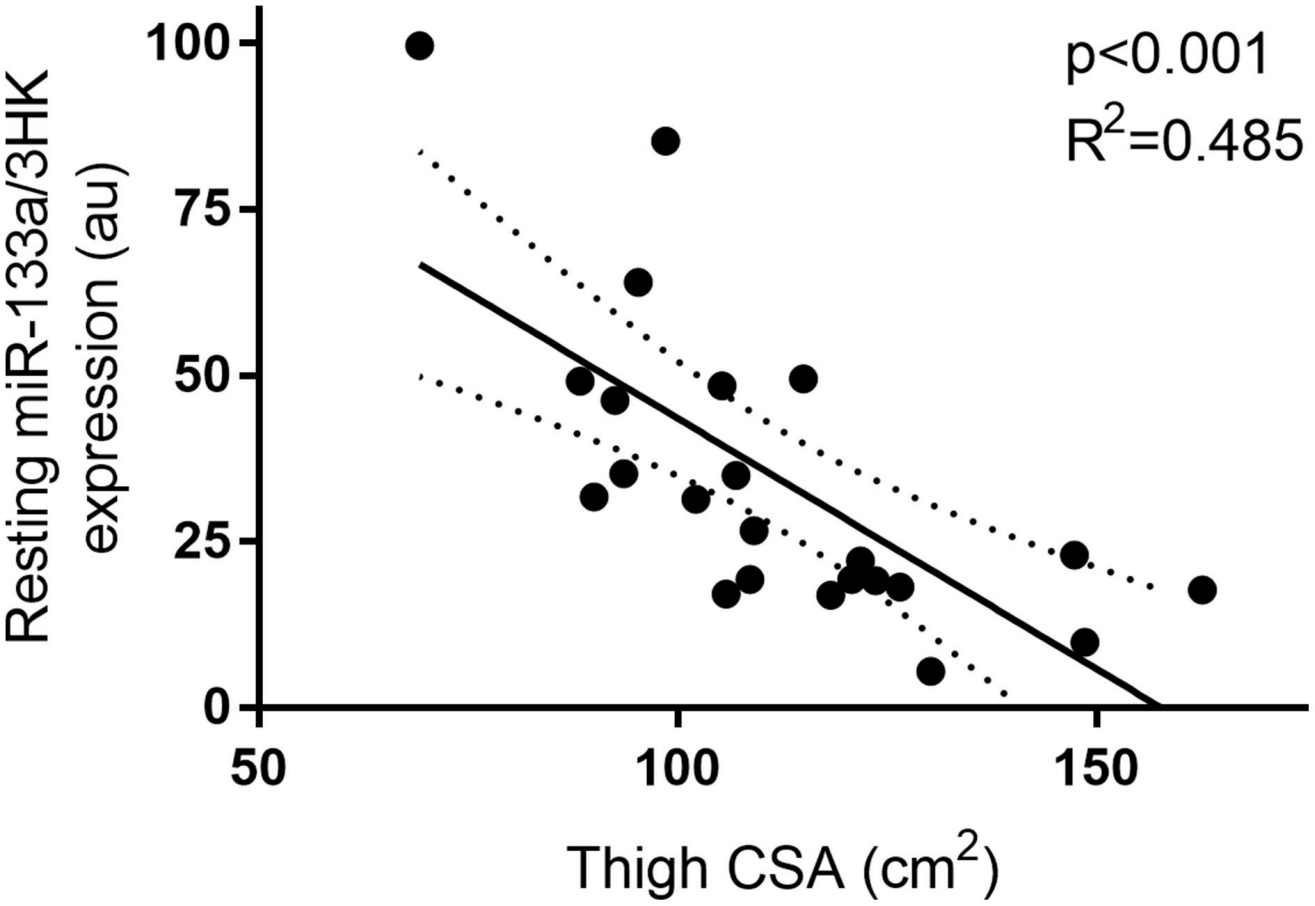
Correlation between resting miR-133a and thigh muscle CSA. miRNA expression is plotted as 2^−Δ*CT*^ on the y-axis with thigh CSA (cm^2^) one the x-axis. The solid line represents the line of best fit as determined by linear regression with 95% confidence intervals.

Furthermore, miR-133a, though unresponsive to exercise and feeding stimuli, demonstrated a negative correlation with thigh CSA (*p* < 0.01 and *R*^2^ = 0.485) at rest (Figure 5).

## Discussion

Of the 19 miRNAs included in the current analyses, six miRNAs (miR-1,-15a,-99a,-148b,-149, and-499a) were differentially expressed between the placebo and increasing doses of 20 and 40 g whey protein supplementation immediately following resistance exercise. The general pattern of expression suggested that protein ingestion following resistance exercise in elderly individuals may result in a miRNA profile similar to what is previously reported in young men (12, 13). When correlation analysis of the changes in miRNA expression relative to the phosphorylation of key kinases required for anabolism was performed, it was observed that miR-208a and-100 both correlated positively with p-P70S6K^Thr389^, whilst miR-206 and-208a expression was positively and negatively correlated with p-Akt^Ser473^, respectively. These data are consistent with the hypotheses recently suggested by Margolis and Rivas (12). Together these findings demonstrate a potential involvement of specific miRNAs in the regulation of hypertrophic signaling events following resistance exercise in human muscle *in-vivo*.

The current study demonstrates a lower muscle miR-1 expression following the ingestion of 40 g whey (containing ~18 g of EAA) group compared to the placebo group at 4 h following exercise. miR-1 expression in the 40 g group however, was not significantly reduced compared to resting pre-exercise levels. The consumption of 20 g of EAA in combination with resistance exercise reportedly reduced miR-1 expression in young but not older individuals (13). These differences in findings between studies suggests that the current population may be more anabolically sensitive than the older participants reported by Drummond et al. (13). However, it has been previously reported that whey protein may be more effective than its constituent EAAs at stimulating an anabolic response (26, 27). The placebo in the present study was non-nutritive so it is not possible to separate the effects of protein *per se* from the energy content of the beverages. However, Drummond's (13) observed age difference in miR-1 following resistance exercise and EAA ingestions in conjunction with the known age-related decrease in anabolic sensitivity suggest protein is a more likely candidate to explain the observed response (6). Whereas, the placebo group appears most similar to the older adults in Drummond et al. Similarly, miR-499a was previously shown to increase 2 h following exercise in elderly but not young participants in the postabsorptive state (9), whilst no change was evident at 6 h following exercise (12). This is congruent with the present finding where miR-499a abundance increased within muscle of placebo treated individuals, a change that was attenuated at 4 h in both the protein supplemented groups when compared to the placebo group. From currently available research it is clear both participant age and feeding status alter the miRNA response to exercise. These findings suggest that whey protein feeding following resistance exercise may help to promote a more youthful post exercise muscle expression pattern of certain miRNAs in older adults. However, the findings are limited by the lack of young adult group and isoenergetic control in the present study.

miR-99a/100 family miRNAs particularly miR-99a and-149 demonstrated a similar pattern whereby expression at 4 h post-exercise was lower in the protein supplemented groups when compared to the placebo group. In trained young men, we previously observed no change in muscle miR-149 following exercise in the postabsorptive state (8). The relatively lower expression of both these miRNAs with protein supplementation in conjunction with a lack of change after fasted resistance exercise in previous studies (8, 9) suggest a role of protein ingestion in regulating miR -99a and -149 abundances. However, this cannot be completely confirmed as these miRNAs have not been measured following post-exercise feeding in a young cohort previously.

Previous *in vitro* work has demonstrated the ability for miRNAs to alter phosphorylation status of several proteins (11, 28, 29). The Akt-mTOR cascade has been extensively studied in response to feeding and resistance exercise with several miRNAs being implicated in the regulation of this pathway however it is unclear if miRNAs can directly modulate phosphorylation status of Akt-mTOR pathway targets (9, 10). In the present study the change in expression of miR-208a was positively correlated with changes in phosphorylation of P70S6K^Thr389^ at 4 h following exercise, explaining ~68% of the observed variance. Expression of miR-499a was also positively correlated with phosphorylation of P70S6K ^Thr389^ at 4 h after exercise, explaining ~72% of participant variability. The positive relationships appear opposite to what is expected based on *in-vitro* models (9, 11). This can be interpreted to support the hypothesis that miRNA may be exerting regulation via negative feedback which is congruent with much of our previous work in muscle (8, 23, 30, 31) and has been proposed in other miRNA models (32–36). Whilst the quantification of p-P70S6K^Thr389^ was limited by the inability to use total P70S6K for normalization, we strongly believe the utilization of total ERK is reflective of the expected pattern. Also, to the best of our knowledge we do not know of any reported changes in total P70S6K or total ERK2 expression upto 4 h following exercise.

miR-208a and-206 are also thought to inhibit Akt signaling upstream of mTOR (10). At 4 h post exercise, miR-206 and miR-208a were positively correlated with p-AKT^Ser473^. These miRs explained ~46% and ~63% of the observed participant variance in p-AKT^Ser473^, respectively. The identified relationships support a likely role of miRNAs in not just transcription and translational inhibition but also in understanding dysregulated signaling pathways that promote anabolic resistance in elderly individuals (9, 10). Although this is a novel finding in human muscle there is indirect evidence from *in vitro* models that miRNAs may be able to influence posttranslational modifications such as phosphorylation (11, 28, 29). It is possible that the relationship between miRNA and protein phosphorylation is the result of a direct interaction via an undescribed mechanism or perhaps more likely miRNAs may regulate protein translation of upstream mTOR effectors which in turn control downstream phosphorylation status. Alternatively, the design of the present study does not preclude the possibility of a simple correlative relationship with no direct mechanistic regulation of phosphorylation by miRNA.

Like miR-99a and-149, miR-148b was down regulated in the protein groups following resistance exercise in comparison with the placebo group. miR-148b is thought to promote Akt signaling via inhibition of PTEN. Further, chronic increases in miR-148b are evident in models of reduced physical activity in rats and humans (30, 37). The present findings suggest that the reduction in miR-148b expression in the protein supplemented groups may act as part of a negative feedback mechanism to promote PTEN dependent inhibition of Akt signaling upstream of P70S6K (38). However, further experiments are required using *in vitro* or transgenic animal models to test this hypothesis.

In agreement with several previous works from our lab and others, miR-133a was found to negatively correlate with thigh muscle CSA at rest (23–25, 30, 39). In the current cohort, a negative relationship was observed between anthropometrically determined thigh muscle CSA and miR-133a expression. The relationship explained ~49% of model variance. This finding is consistent with several studies in rested middle aged men (24, 25), healthy controls vs. competitive powerlifters (23), and the patterns seen with limb immobilization (30), as well as following overload induced hypertrophy via surgical ablation in rats (39). From our previous work, miR-133a in combination with miR-146a explained ~33% and ~34%, respectively, of participant variability in whole body and leg lean mass, respectively. The relationship observed in the current study was stronger than previously reported, which could be a random effect due to the smaller sample size or may reflect a greater importance of miR-133a in aged muscle.

The current study was limited by the differences in energy contents of the beverages provided to each group, making it impossible to conclusively attribute the results to whey protein *per se* rather than the small differences in energy intake associated with the protein. This is further pronounced in the placebo group who were asked to perform exercise following an overnight fast and not provided with any post exercise nutrition. However, given p-Akt abundances were not differentially regulated between supplement groups, it is unlikely that observed difference in miRNA response were related to insulin signaling (40) as might have been expected if energy intake was the dominant mechanism. The lack of a young control group prevents definitive conclusions about age related responses, thus any interpretation of the current results concerning the age-related differences in anabolic sensitivity can only be cautiously inferred from existing literature. Further, the lack of sufficient remaining muscle tissue prevented the measurement of targets regulating muscle catabolic processes which could have added additional depth to the results.

## Conclusion

The present study identified a clear effect of large dose whey protein supplementation on post exercise muscular miRNA expression patterns. Additionally, the reported miR-206,-208a, and-499a expression changes demonstrated strong correlations with changes in expression of p-P70S6K^Thr389^ and p-Akt^Ser473^ at 4 h following exercise. These findings strongly implicate miR-206, 208a, and -499a in the regulation of phosphorylation of these proteins following exercise in elderly men. The pattern of expression suggests that whey protein ingestion in elderly men in combination with exercise more closely mimics the post-exercise microRNA response evident in younger adults. These results also for the first time demonstrate a relationship between changes in the abundances of p-P70S6K^Thr389^ and p-Akt^Ser473^ and alteration in miRNA expression following exercise *in-vivo*. The identified pattern suggests a potential role for miRNAs as negative feedback regulators of this key anabolic signaling cascade, which requires further study. The current findings suggest the need for more mechanistic knockout and *in-vitro* models to better understand the role miRNAs play in modulating the acute post-exercise anabolic response in the presence of whey protein and other nutrition sources. Furthermore, similar studies in individuals with low muscle function may also be required to better validate the ability of whey protein supplementation in improving anabolic signaling responses in elderly individuals. Lastly, the results presented also provided additional support for the role of resting miR-133a expression as a biomarker or possible causative agent in the control of muscle size.

## Ethics Statement

This study was carried out in accordance with the recommendations of Deakin University Human Research Ethics Committee' with written informed consent from all subjects. All subjects gave written informed consent in accordance with the Declaration of Helsinki. The protocol was approved by the name of committee.

## Author Contributions

RD, CM, and DC-S designed the study and wrote the manuscript. RD, NZ, JM, and VF performed experiments. JM, AP, and PD sample collection. RD, JM, NZ, CH, AP, PD, DC-S, and CM analyzed data. CM is responsible for the final content of the manuscript. All authors critically evaluated and approved of the final content of the manuscript.

### Conflict of Interest Statement

The authors declare that the research was conducted in the absence of any commercial or financial relationships that could be construed as a potential conflict of interest.
